# Society Meetings

**Published:** 1915-12

**Authors:** 


### SOCIETY MEETINGS

**Brief reports and announcements of meetings of societies of Western New York, and those of general scope, are requested from Secretaries. Copy should be on hand the fifteenth of the month. Full transactions will be published at cost of composition.**

**DOCTOR: Is your Society properly represented here? If not, please bring our standing announcement to the attention of the members.**

**The American College of Surgeons,** at its recent meeting in Boston, admitted 450 additional fellows. The officers were all re-elected. The endowment fund, by the addition of about $100,000, is close to half a million.

The regular monthly meeting of the **Elmira Academy of Medicine** was held November 3. Scientific program: Ectopic Gestation, Charles A. Bentz; Surgical Treatment of Ectopic Gestation, Ross G. Loop. A collation was served.

**The American Society for the Study of Alcohol and Other Narcotics** will hold its 45th annual meeting at Washington, D. C., December 15 and 16. This was the first society of medical men in the world to take up the scientific study of alcohol and other narcotics. Its papers and transactions have been published in the Journal of Inebriety, and comprise the first scientific literature on this subject. 31 papers will be read at this meeting by specialists and distinguished medical and scientific men. These studies will be confined exclusively to the effects of alcohol and drugs on the body and brain, based on clinical experience and laboratory observations.

The public are cordially invited to be present. Programs can be had by addressing the Secretary, Dr. T. D. Crothers, Hartford, Conn.

At the 46th annual meeting of the **American Medical Editors Association,** New York, October 18 and 19, Dr. Edw. C. Register of Charlotte, N. C., was elected president; Dr. W. A. Jones of Minneapolis, vice-president; Dr. G. M. Piersol of Philadelphia, vice-president; Dr. J. MacDonald, Jr., of New York, secretary-treasurer.

At the annual meeting of the **Detroit Academy of Medicine,** held October 12, the following officers were elected: President, Dr. Charles D. Aaron; Vice-president, Dr. Guy Connor; Secretary and Treasurer, Dr. Alpheus F. Jennings.

---

The annual meeting of the **Ontario County Medical Society** was held at Canandaigua October 19. The following officers were elected for the ensuing year: B. T. McDowell, Bristol Center, president; C. W. Selover, Stanley, vice-president; D. A. Eiseline, Shortsville, secretary and treasurer. The following papers were read: Cancer Prophylaxis, Donald Guthrie, M. D., Sayre, Pa.; Fracture of the Skull, with report of a case, Charles W. Webb, M. D., Ithaca.

---

**The National Society of Keep Wells** has been organized, with Mrs. Arthur MacDonald of Washington, President; Mrs. Mary Stevens Beall, Secretary, and Miss Dorothy Dent, Treasurer. Semi-monthly meetings are held and popular lectures are given. It is intended that chapters should be formed in various cities of the country with the idea of furthering a knowledge of personal and domestic hygiene and prophylaxis of disease among women. We take pleasure in endorsing this movement and trust that such local branches may be established in our territory, and that physicians will call the attention of their wives to this movement.

---

**The Medical Society of the County of Niagara,** at its November meeting, elected the following officers: President, H. A. Wilmont, Middleport; Vice-president, John C. Plain, Ransomville; Secretary, L. R. Hurlburt, Lockport; Treasurer, Wm. A. Scott, Niagara Falls.

---

**The Rochester Academy of Medicine** met under the auspices of Section III, November 10. Dr. David B. Jewett gave a paper on Diagnostic Problems in Abdominal Diseases of Children; Dr. Joseph Roby on A Phase of Gastro-Enteritis in Children.

---

**The Buffalo Academy of Medicine** has held the following meetings since our last report: October 27, Pathologic Section, Reactions and Results of Treatment in Cerebro-spinal Syphilis, George Draper of New York, discussion opened by James Wright Putnam.

November 3, Surgical Section, Exhibition of Cases illustrating the Radical Treatment of Pott's Paraplegia by the Albee Bone Graft, Prescott Le Breton; Research Work in the Surgery of the Duodenum, with Specimens, Edgar R. McGuire, X-ray slides by E. C. Koenig.

November 10, Medical Section, Ferments, N. R. Kavinoky; Some Unusual Features of Empyema, John H. Pryor; Formation of Gas in Stomach, A. L. Benedict.

November 17, Section of Obstetrics and Gynaecology, Symposium on Infant Welfare; What the Health Department is Doing, Dr. Arthur C. Schaefer; Health Department Obstetrical Clinic, Dr. Irving S. Potter; University of Buffalo Obstetrical Clinics, Dr. E. A. D. Clarke; Prevention of Infant Blindness, Dr. Park Lewis; Babies Milk Dispensaries History, Dr. N. G. Russell; Present Work, Dr. Carl G. Leo-Wolf.

